# Facial Weakness, Diplopia, and Fever in a 31-Year-Old: An Atypical Case of Tuberculous Meningitis

**DOI:** 10.7759/cureus.1918

**Published:** 2017-12-07

**Authors:** Ahmed H Qavi, Tasnim F Imran, Zachariah Hasan, Fariha Ilyas

**Affiliations:** 1 Department of Medicine, Albert Einstein College of Medicine, Montefiore Medical Center, New York, United States; 2 Department of Medicine, Brigham and Women’s Hospital, Harvard Medical School, Boston, MA, United States; 3 Department of Medicine, Eastern Virginia Medical School, Norfolk, VA, United States; 4 Department of Medicine, Northwell Long Island Jewish Medical Center, New York, United States

**Keywords:** tbm, meningitis, tuberculous meningitis, infectious diseases, cranial nerves

## Abstract

Tuberculous meningitis (TBM) is an infection of the central nervous system (CNS) meninges that carries high morbidity and mortality. It is important to recognize, as patients may present with atypical symptoms. We describe the case of a 31-year-old man with a history of diabetes who presented with a sub-acute onset of right-sided facial weakness and right gaze difficulty with diplopia. History revealed low-grade fever, right-sided headache, fatigue and moderate weight loss for the past several weeks. The patient did not report neck stiffness, rigidity, fever, chills or cough. The physical exam revealed sixth nerve palsy with a right Horner’s syndrome. Magnetic resonance imaging (MRI) of the brain showed pachymeningeal enhancement. A spinal tap revealed elevated white blood cells (WBCs), glucose and protein; cerebrospinal fluid (CSF) culture showed Mycobacterium tuberculosis. The patient was diagnosed with TBM and treated with isoniazid, rifampin, pyrazinamide, ethambutol and vitamin B6 for 12 months.

The timely diagnosis of TBM can be challenging due to a nonspecific clinical presentation. In patients with a sub-acute onset of headache, fever and meningeal signs, TBM should be considered in the differential. If suspected, treatment should be initiated immediately to prevent further neurological impairment and death.

## Introduction

Tuberculous meningitis (TBM) is the most lethal type of extrapulmonary tuberculosis and is especially severe in immunocompromised patients [1-2]. Patients typically present with malaise, apathy, mild fever, long-lasting headache with rising intra-cranial pressure, vomiting, focal neurological deficits and confusion [2]. TBM is usually diagnosed on the basis of clinical evidence that is combined with neuroimaging abnormalities, laboratory findings and cerebrospinal fluid changes (increased protein, low glucose and mononuclear cell pleocytosis) [2]. The disease is treated with anti-tuberculosis therapy. A delay in starting treatment is proportional to an increased risk of mortality or survival in a stupor, coma or hemiparesis [1]. Here, we report a case with an atypical presentation of TBM in a diabetic patient.

## Case presentation

A 31-year-old man with a history of diabetes presented with a right-sided facial weakness for three weeks. He reported low-grade fever, right-sided headache, tiredness and fatigue for the past month. The headache was dull, bilateral, had a gradual onset and was persisting with the same severity. There was no history of photophobia, neck stiffness, rigidity, nausea, vomiting or any kind of aura. He reported moderate weight loss four months ago. The patient did not have chills, cough or other illnesses. His past medical history was positive for diabetes, diagnosed nine months ago. His social history revealed habitual smoking. He had travelled to India nine months ago but denied any recent illnesses.

On physical examination, the patient appeared to be weak but not cachectic. He had restricted eye movement (sixth nerve palsy) on the right side. The pupils were round and reactive to light (the right slower than the left) with a mild Horner’s syndrome on the right. No nystagmus was seen. A lower motor neuron lesion resulting in right-sided facial weakness was also noted. The exam revealed no paralysis, sensory loss or tremors. The patient was oriented to time, place and person. A chest examination showed a palpable mass on the right side. Breathing sounds were normal.

**Figure 1:**
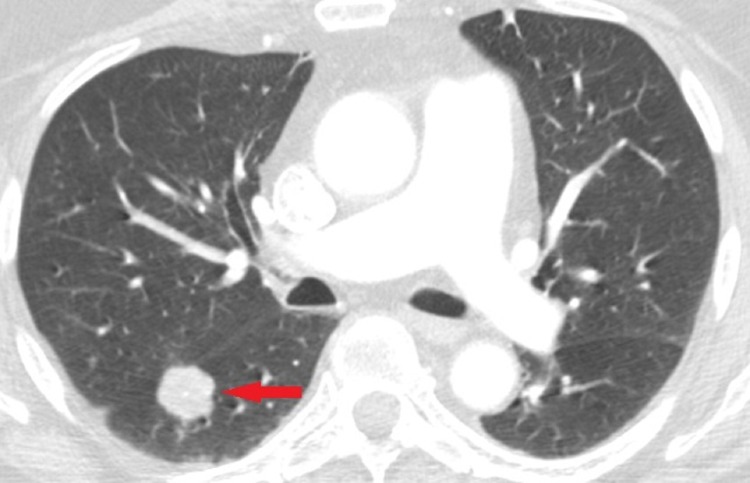
CT chest showing a benign right lower lung mass CT: computed tomography

A laboratory examination indicated normal white blood cells (WBC), platelets and elevated glucose (160). A spinal tap revealed elevated WBC (10), glucose (120) and protein (66). Given that the patient had a mild Horner’s syndrome, a computed tomography (CT) scan of the chest was obtained to assess for masses such as a Pancoast tumour. A CT scan of the chest revealed a benign right lower lung mass, lymphadenitis and hepatomegaly (Figure 1).

No signs of enlarged heart, pleural/pericardial effusion or nodules were noted. A microscopic examination of the sub-cranial lymph node smear showed rare, ciliated, reactive bronchial epithelial cells and histiocytes. The microscopy of the right lower lung lobe revealed mixed inflammatory cells along with alveolar macrophages, reactive bronchial cells and squamous cells. The differential included infectious diseases, such as tuberculosis, cryptococcal, Lyme disease, vasculitis, lymphoma and malignancy.

**Figure 2:**
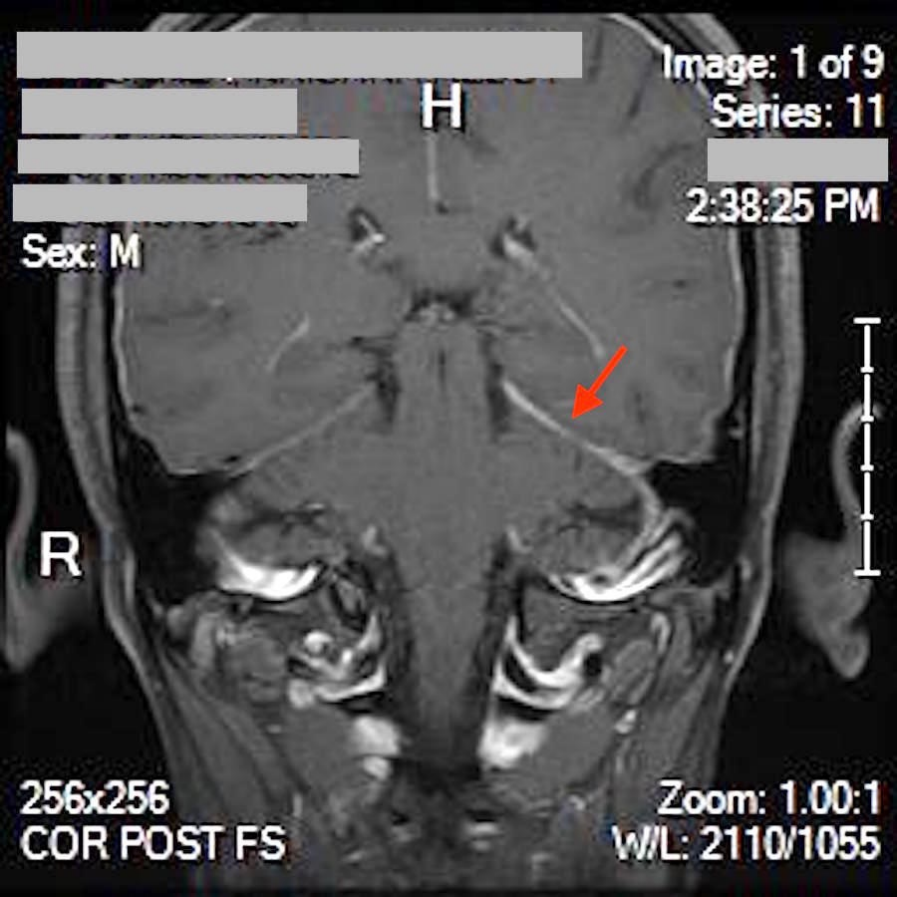
MRI brain without contrast, showing asymmetric tentorial enhancement (red arrow) MRI: magnetic resonance imaging

Magnetic resonance imaging (MRI) of the brain was obtained due to neurological deficits. It showed pachymeningeal and diffuse homogenous dural enhancement along with asymmetric tentorial enhancement, which is an uncommon finding in TBM (Figure 2). 

The differential diagnosis thought out based on the MRI included an inflammatory, infectious or (less likely) neoplastic process. Clinical correlation was advised to exclude the possibility of mycobacterial disease. The patient underwent an MRI of the brain and cranial nerves with gadolinium, which was normal pre- and post-gadolinium. A cerebrospinal fluid (CSF) culture showed Mycobacterium tuberculosis, and the patient was subsequently diagnosed with tuberculosis meningitis.

The patient was immediately started on anti-tubercular therapy. He was inducted on isoniazid, rifampicin, pyrazinamide and ethambutol for the first 2 months. Dexamethasone was also added to the treatment as adjunctive therapy. The patient’s fever subsided after 3 weeks. His follow-up MRI after 2 months showed no significant changes. His CSF analysis was, however, improved. Isoniazid and rifampicin were continued for another 10 months. His MRI at 10 months showed complete resolution of the abnormal enhancements.

## Discussion

TBM is the most lethal form of tuberculosis [1-2] and those surviving are often left with severe neurological disabilities [2]. A central nervous system (CNS) infection with M. tuberculosis results in a granulomatous, inflammatory response that involves the meninges, cisterns and parenchyma [3]. Retrospective studies show that about 75% of individuals have a tuberculous infection at least one year prior to admission for meningitis [1]. Alcoholism and other chronic, immune-suppressing debilitating diseases increase the risk of developing TBM [2].

TBM is not always an easy diagnosis [4]. Sometimes, the CSF culture and, quite frequently, the chest x-ray in a TBM patient comes out negative but this should not exclude the disease and treatment should be initiated quickly and empirically based on the history and presentation [4]. Untreated TBM is fatal 10 out of 10 times [4]. One 51-year-old man presented with mental status changes, ophthalmoplegia, gait ataxia and history of alcohol use, shooting arrows at Wernicke’s encephalopathy. The patient had no neck stiffness, vomiting or headache. The CSF showed an atypical neutrophilic predominance. It was only the persistent fever that included TBM in the differential [5]. Sometimes, TBM can manifest with behavioural changes while other cases report patients with seizures and hemiparesis [6]. Our patient presented with a sub-acute headache and right-sided facial weakness without the presentation of any neck stiffness, vomiting or confusion.

TBM remains a challenging diagnosis with subtle and various clinical presentations. Zhang et al. described a patient presenting with fever, headache and facial numbness. The MRI scan showed isointense T1 and hyperintense T2 lesions in the dorsal medulla and cervical spine, mimicking a demyelinating disease. CSF parameters were normal, except for a high WBC count (lymphocytic predominant 80%) and negative for Mycobacterium tuberculosis and acid-fast bacilli (AFB) staining. TBM was suspected due to the patient’s long-term clinical course and close TB contacts, and he was started on an anti-tuberculosis treatment (ATT) regimen. The patient subsequently had a gradual resolution of symptoms [7].

Carrilo et al. described a patient with prior history of migraines presenting with sudden onset of frontoparietal headaches, nuchal rigidity and faciobrachial hemiplegia. Initial CT findings showed parieto-temporal hypodense lesions and MRI studies revealed a middle cerebral artery (MCA) infarct in the parieto-temporal region. Progressively worsening mononuclear pleocytosis and hyperproteinemia suggested a diagnosis of TBM and prompted the initiation of ATT. The patient had rapid improvement of headaches and CSF parameters, however, the MCA infarct persisted [8].

An incidence of visual impairment has been reported in cases of TBM, causing significant morbidity. Verma et al. reported a rare occurrence of bilateral primary optic atrophy in a patient with a history of fever and headaches associated with vomiting and decreased vision bilaterally. CSF findings revealed elevated proteins and lymphocyte count, and the MRI showed lepto-meningeal enhancement. Optical coherence tomography {OCT) was performed and showed decreased retinal fibre thickness bilaterally in the superior and inferior quadrants. ATT was begun, however, the patient’s visual acuity did not improve during subsequent follow-up. Verma et al. described another case of optic atrophy in a patient with prior history of ATT that was treated symptomatically, with no improvement in visual acuity either [9].

Timely diagnosis can be difficult due to nonspecific clinical presentations, as described above [1-2]. A high clinical index of suspicion is required to diagnose TBM, and mortality is high if left untreated [10]. The gold standard for the diagnosis of TBM is the growth of M. tuberculosis from the CSF [2]. However, it takes four to eight weeks for the cultures to become positive and up to 45% of patients with suspected TBM have negative cultures [10]. Thus, the application of multiplex PCR is of high value, as this test increases the sensitivity and specificity of the diagnosis of TBM and is less time-consuming [3]. However, PCR is not readily available. 

The onset of TBM is usually gradual and the disease course is typically divided into three stages. In the beginning, it is characterized by behavioural changes, irritability, anorexia, and apathy [3]. If left untreated, TBM progresses into its second stage with increased intracranial pressure, resulting in drowsiness, neck stiffness, cranial nerve palsies, vomiting and convulsions. Ultimately, the disease culminates in the third stage, which is depicted by coma, irregular pulse and respiration, stupor and abnormal posturing due to severe brain injury and death [2].

In this case, it was challenging to obtain the diagnosis of TBM since the patient did not present with typical symptoms. This was a rare presentation of the disease. He had an abrupt onset of right-sided facial weakness, 6^th^ nerve palsy, diplopia and a mild Horner syndrome on the right side. The patient’s multiple cranial neuropathies were not accompanied by any neck stiffness, rigidity, focal weakness, paralysis or tremors to suggest the possibility of TBM. His symptoms of low-grade fever, headache and fatigue could have been attributed to a common cold, flu or a non-specific headache. The MRI findings were inconclusive.

The CSF glucose was elevated (120), whereas the typical value in TBM is less than 45 mg/dL. CSF protein was also only mildly elevated (66), which is low as compared to the range of 100 to 500 mg/dL in most patients. The present case reveals the rarity and diversity of the clinical manifestations of TBM. Therefore, it is essential to have a high clinical suspicion when a patient presents with acute neurological deficits.

## Conclusions

Patients who present with a sub-acute onset of headache, fever and meningeal signs should be suspected for having TBM. ATT should be commenced immediately even if a culture confirmation of the presence of Mycobacterium tuberculosis is not available because the outcome of TBM largely depends on the degree of neurological impairment at the time of initiation of the therapy. Hence, when diagnosed early and treated appropriately, the prognosis of TBM is much improved. This report is intended to increase clinician awareness of late and atypical presentation, and emphasize the importance of the early diagnosis of TBM.
